# Plasmonic Hot-Carrier Generation and Catalysis in Ti_3_C_2_O_2_ from Real-Time TDDFT Simulations

**DOI:** 10.1021/acs.nanolett.6c00650

**Published:** 2026-04-10

**Authors:** Na Zhang, Shoutian Sun, Bin Wang

**Affiliations:** † School of Sustainable Chemical, Biological and Materials Engineering, 6187 University of Oklahoma, Norman, Oklahoma 73019, United States; ‡ Department of Chemical and Biological Engineering, Tufts University, Medford, Massachusetts 02155, United States

**Keywords:** Ti_3_C_2_O_2_, plasmonic catalysis, hot carrier, time-dependent density functional theory (TDDFT), localized surface plasmon resonance (LSPR), CO_2_ reduction

## Abstract

Photoinduced hot electrons are central to plasmon-driven catalysis. Atomically thin Ti_3_C_2_O_2_, with high carrier density and broad optical absorption, offers a promising platform for plasmon-driven reactions. However, comprehensive investigations of its plasmon resonance, hot-carrier generation, and plasmonic catalytic performance remain limited. In this work, real-time time-dependent density functional theory (rt-TDDFT) was employed to study Ti_3_C_2_O_2_’s plasmon excitation and hot-carrier generation from nonradiative plasmon damping. The temporal evolution of the dipole moment reveals plasmon resonance in Ti_3_C_2_O_2_, followed by strong plasmon damping that redistributes the stored energy to generate hot carriers. Ti_3_C_2_O_2_ with low oxygen vacancy concentration (O_v_-Ti_3_C_2_O_2_) exhibits plasmonic behavior resembling the pristine surface, and the plasmon-generated hot electrons can markedly reduce the dissociation barrier of CO_2_ at the oxygen vacancy. These findings provide fundamental insights into the plasmonic properties of Ti_3_C_2_O_2_ and how they drive its catalytic performance in surface reactions, which is valuable for advancing plasmon-driven catalysis.

## Graphical abstract

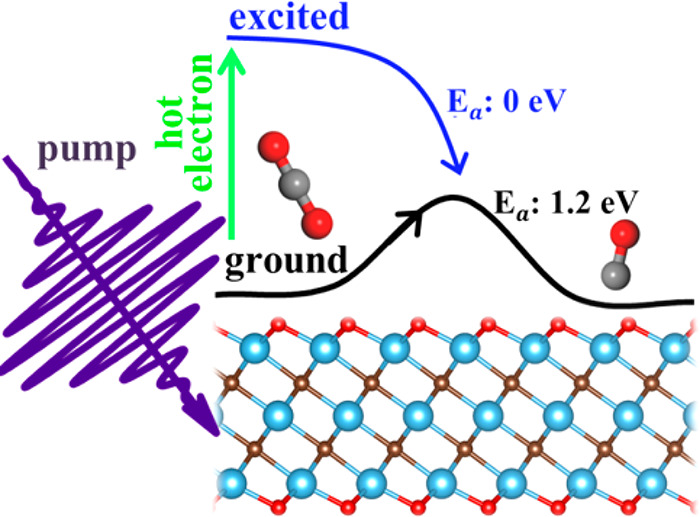

Plasmon-driven catalysis harnesses light to efficiently promote chemical reactions, offering a distributed, environmentally friendly approach for sustainable chemical processes. Plasmon-mediated reactions are enabled by localized surface plasmon resonance (LSPR), which originates from the collective oscillation of free electrons in plasmonic nanostructures.^1–4^ LSPR can be used to modulate reaction rates and product selectivity through distinct mechanisms arising from its interaction with surface-adsorbed species.^5–12^ Highly energetic (or “hot”) charge carriers generated by the nonradiative damping of LSPR play a key role in plasmon-mediated processes.^13^ In addition to Landau damping, plasmon decay into hot carriers can also occur via interband transitions, phonon- or impurity-mediated intraband transitions, and electron–electron (Umklapp) scattering-assisted processes.^14–18^ These initially nonthermalized charge carriers redistribute their energy through electron–electron scattering (EES), leading to a quasi-thermalized state at a temperature higher than the lattice temperature.^19^ Prior to coupling with the lattice vibrations and generating heat, the generated hot carriers can transfer to frontier orbitals of surface-adsorbed molecules either directly or indirectly, depending on whether they undergo EES process before injection, thereby facilitating chemical reactions. These investigations have largely focused on prototypical metal nanoparticles such as Au, Ag, and Cu due to their strong plasmonic response under irradiation of visible light, providing valuable insights into LSPR damping mechanisms and the role of LSPR in facilitating catalytic reactions.^20–27^

The metallic MXene Ti_3_C_2_T_*x*_ (with typical surface group T_*x*_ including O, OH, F, and Cl) has emerged as a novel plasmonic material, due to its high free-electron density and plasmon features spanning the visible to infrared range.^28–30^ Unlike conventional metallic nanomaterials such as Au, Ag, and Cu, its ultrathin layered structure effectively suppresses energy loss of nonthermalized electrons, thereby enabling unique interaction of these energetic electrons with surface-adsorbed molecules. It was proposed that nonthermalized electrons generated by LSPR decay in Ti_3_C_2_T_*x*_ can transfer directly to surface-adsorbed methylene blue on a ∼50 fs time scale, bypassing the EES process.^31^ Direct transfer avoids significant energy loss, allowing nonthermalized hot electrons to populate specific unoccupied orbitals, thereby achieving precise energy matching and improving selectivity of desired products. In traditional plasmonic metals such as Au, Ag, and Cu, the generally inert surfaces lead to weak molecular adsorption, and therefore such direct transfer pathway is rather challenging. Moreover, wavelength-selective excitation of plasmon modes in Ti_3_C_2_T_*x*_ enables activation of both edge and in-plane catalytic sites, thereby enhancing their efficiency in hot-carrier-mediated water splitting.^32^ Beyond its intrinsic properties, Ti_3_C_2_T_*x*_ can form a heterostructure with another photoresponsive material, such as MoS_2_, that exhibits a unique secondary excitation and ultrafast charge/energy transfer, offering new insights for photochemical and optoelectronic applications.^33^ Despite advances in studying plasmonic Ti_3_C_2_T_*x*_,^34–43^ a theoretical understanding of its fundamental plasmonic property, hot-carrier generation from LSPR decay, and their catalytic roles remain limited.

In this work, we employed real-time time-dependent density functional theory (rt-TDDFT) simulations to investigate the optical properties, plasmon response to incident light, and hot-carrier generation in Ti_3_C_2_O_2_. The absorption spectrum and plasmonicity index exhibit multiple plasmon resonances spanning the near-infrared to near-ultraviolet regions. The infrared plasmon resonance agrees well with previous experimental results. Within the dipole approximation, the strong dipole moment reflects the collective response of free electrons in Ti_3_C_2_O_2_ to the laser pulse. Hot-carrier generation from LSPR damping is investigated together with a transition contribution map (TCM) to visualize the transition intensities between holes and electrons. To study the catalytic role of hot carriers in Ti_3_C_2_O_2_, we designed a defective Ti_3_C_2_O_2_ surface containing an oxygen vacancy (O_v_). The absorption spectrum of O_v_-Ti_3_C_2_O_2_ closely matches that of pristine Ti_3_C_2_O_2_. Our results show that CO_2_ undergoes polarization-induced activation at the O_v_ site, accompanied by a downward shift of its energy levels relative to free CO_2_. The downward shift of the lowest unoccupied molecular orbital (LUMO) of adsorbed CO_2_ may host the hot electrons excited from the occupied states near the Fermi level of O_v_-Ti_3_C_2_O_2_, thereby facilitating dissociation of CO_2_ into CO. The linear expansion ΔSCF results reveal that hot electrons can eliminate the kinetic barrier for CO_2_ dissociation, thereby enhancing the conversion process. These findings provide fundamental insight into the plasmonic properties of Ti_3_C_2_O_2_ and highlight the valuable role of hot carriers in driving chemical reactions.

Figure 1a shows the atomic structure of Ti_3_C_2_O_2_, where a 5 × 5 × 1 supercell is employed to investigate its plasmonic properties (see Figures S1 and S2 for convergence tests). The unfolded band structure (Figure S3) reveals the metallic nature of Ti_3_C_2_O_2_, contributing to its plasmonic behavior.^31^ As shown in Figure 1b, we first investigated the photoabsorption spectrum of pristine Ti_3_C_2_O_2_ using the δ-kick technique (the computational details are provided in the Supporting Information). The spectrum reveals multiple plasmon resonances with different collective characteristics spanning from the near-infrared to the near-ultraviolet regions, with pronounced peaks at 1.51 and 3.34 eV. The plasmonicity index quantifies the collective (plasmonic) character of absorption features.^44^ As shown in Figure 1b, the 1.5 eV mode is predominantly plasmonic, whereas the 3.34 eV mode shows a more mixed character with single-particle contributions (see the Supporting Information for more details). Ti_3_C_2_T_*x*_ has been reported to exhibit plasmon resonances at ∼1.50 eV in near-infrared region and at ∼ 3.1 eV in near-ultraviolet region.^28–31,45–48^ Our calculated results are in good agreement with the experimental observation. To excite the LSPR in the Ti_3_C_2_O_2_, a monochromatic ultrafast Gaussian light pulse is applied perpendicularly to its surface along the *z* axis. The light pulse is described by the electric field ε­(*t*) = ε_0_ cos­[ω_0_(*t* – *t*_0_)] exp­[−(*t* – *t*_0_)^2^/τ_0_^2^]. The central frequency of this pulse is 3.34 eV. It is centered at 10 fs, with a duration time of 3 fs. As shown in Figure 1c, a peak electric field strength ε_0_ of ∼510 μV/Å was used, to ensure that the response of Ti_3_C_2_O_2_ stays within the linear regime. The plasmonic response of Ti_3_C_2_O_2_ to this light pulse is described by the dipole approximation.^49,50^ As shown in Figure 1c, the application of a light pulse to Ti_3_C_2_O_2_ induces a dipole moment response, with the strongest electron-density oscillations appearing at ∼10 fs. The dipole moment exhibits a two-stage damping behavior, the oscillation amplitude decreases rapidly by ∼15 fs and then continues to decay more slowly from 15 to 30 fs (see the inset of Figure 1c), becoming nearly damped within the 30 fs propagation window. This temporal evolution highlights the intrinsic instability of collective electron motion, which undergoes dephasing through multiple channels. Benchmark rt-TDDFT simulations of Ag_20_ and Au_20_ (Figure S4) show that, under identical field parameters at the respective plasmon resonances, Ti_3_C_2_O_2_ displays much faster dipole-moment damping than both clusters. The microscopic origin is likely complex due to its sensitivity to the dimensionality, size, and electronic structure and will be a subject of separate study. The rapid plasmon damping in ultrathin layered Ti_3_C_2_O_2_ has been reported to the large imaginary part of its dielectric function that leads to ultrastrong light absorption.^35^ Consequently, the plasmon lifetime is shortened, while hot carriers are generated quickly via nonradiative damping, which benefits photocatalysis as discussed later in this work.

**Figure 1:**
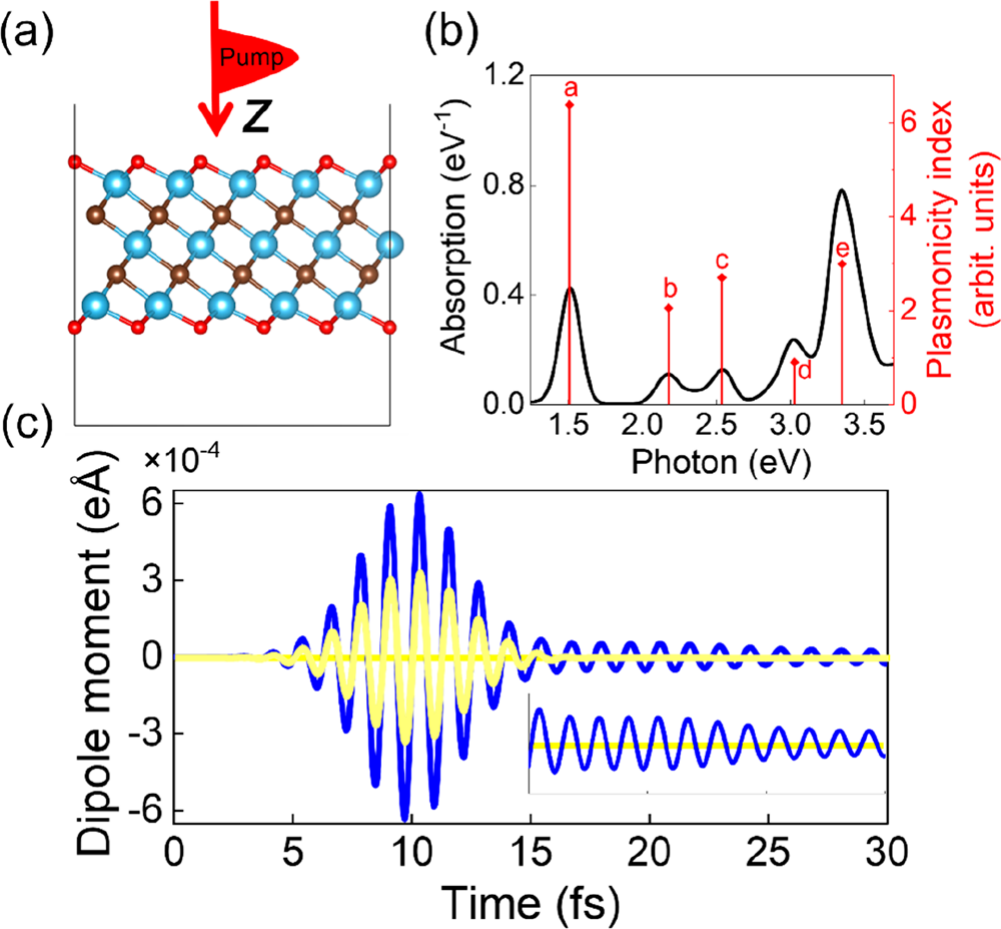
(a) Atomic structure of Ti_3_C_2_O_2_. The red, brown, and light-blue spheres represent O, C, and Ti atoms, respectively. (b) Optical absorption spectrum of Ti_3_C_2_O_2_ (black) and the corresponding plasmonicity index (red diamonds; vertical lines mark the peak positions). (c) Electric field pulse applied to induce the plasmon resonance in Ti_3_C_2_O_2_ (yellow curve) and the plasmon response featured by the temporal evolution of dipole moment (blue curve). The inset shows a magnified view of the dipole response from 15 to 30 fs. The δ-kick infinite-frequency pulse (b) and the single-frequency pulse (c) applied perpendicularly to the Ti_3_C_2_O_2_ surface along the *z* axis.

Upon absorbing light, Ti_3_C_2_O_2_ stores the energy from incident light in its excited state, which initially sustains collective electron oscillations and subsequently contributes to hot-carrier generation through plasmon nonradiative decay. As shown in Figure 2, the hot-carrier occupation and the electron–hole transition intensity increase over time. At 10 fs, the absorbed energy primarily drives coherent collective oscillations, with electron–hole pairs remaining strongly bound by Coulomb interactions.^51,52^ Consequently, the electron–hole transition intensity is weak, and the hot-carrier occupations remain low, while the collective motion is short-lived and decays through multiple channels. As the plasmon decays, the energy initially stored in the collective electronic response is progressively converted into single-particle excitations by nonradiative damping, leading to hot-carrier generation. From *t* = 10 to 15 fs, the TCM intensity increases, indicating strengthened electron–hole excitations, accompanied by a gradual rise in hot-carrier occupations. Beyond ∼15 fs, the TCM pattern changes weakly within the 30 fs propagation window, suggesting that the dominant electron–hole transition has largely saturated. Overall, the time-dependent growth of electron–hole transition provides direct evidence of ultrafast dephasing and energy transfer from the initial collective response into hot-carrier generation in Ti_3_C_2_O_2_.

**Figure 2:**
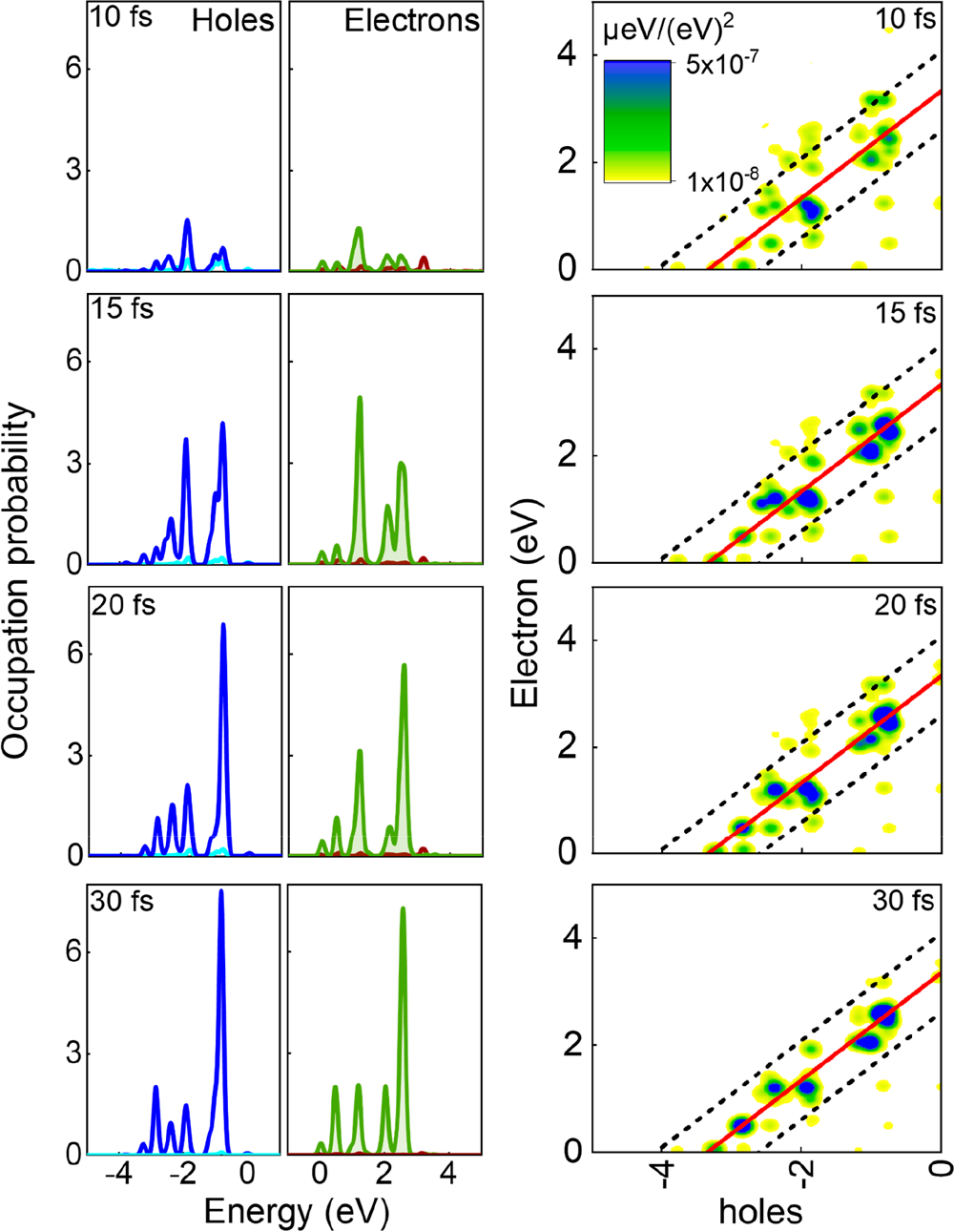
Left panel: Occupation probabilities of holes and electrons at 10, 15, 20, and 30 fs. The cyan curve in the hole panel and the red curve in the electron panel denote the occupations originating from nonresonant transitions. Right panel: TCMs at 10, 15, 20, and 30 fs. The solid red line indicates the central frequency (ω_0_ = 3.34 eV), while the black dashed lines denote the pulse bandwidth (ω_0_ ± 2σ, where σ = √2/τ_0_).

As illustrated in Figure 3a, the TCM at 30 fs and the hot-carrier energy distribution (Figure 2) reveal that the dominant electron–hole transitions in Ti_3_C_2_O_2_ are concentrated between −0.82 and +2.52 eV (relative to the Fermi level), where both transition intensity and hot-carrier population reach their maximum. Figure 3b shows the spatial distribution of these energetic carriers at 30 fs, which indicates that hot electrons are predominantly localized on Ti-derived unoccupied orbitals, whereas hot holes are mainly localized on C-derived occupied orbitals, consistent with the projected density of states (pDOS) in Figure 3a. These hot carriers are generated at or near the surface, and the ultrathin layered structure of Ti_3_C_2_O_2_ potentially shortens the diffusion distance of these hot carriers to surface, thereby reducing energy loss and enhancing their effectiveness in driving chemical reactions.

**Figure 3:**
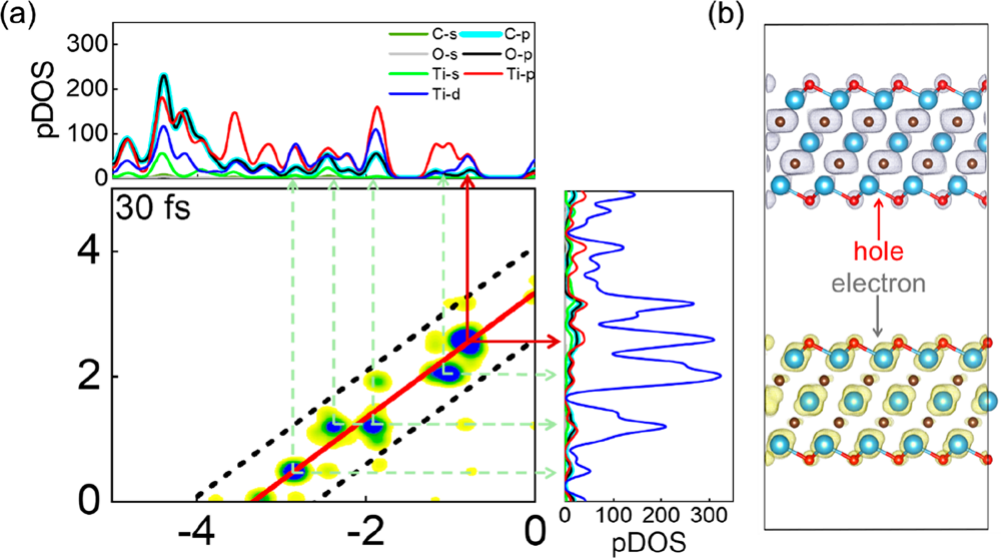
(a) TCM at 30 fs together with the pDOS, illustrating the transition intensities and the energy locations of electrons and holes. The arrows indicate electron–hole transitions, with the red solid arrows highlighting the dominant transition. (b) Spatial distribution of hot carriers in Ti_3_C_2_O_2_, where yellow and gray denote electrons and holes, respectively.

Here we use CO_2_ reduction as the probing reaction. We previously studied the same reaction on Cu_2_O, which is integrated with a plasmonic material, such as Al, to harvest the solar energy.^53,54^ We anticipate the Ti_3_C_2_O_2_ can play both a plasmonic and catalytic role. To facilitate CO_2_ dissociation into CO on Ti_3_C_2_O_2_, we introduced an oxygen vacancy (4% vacancy concentration), forming O_v_-Ti_3_C_2_O_2_ as a prior work suggested the O_v_ site on Ti_3_C_2_O_2_ could serve as the active site for CO_2_ reduction.^55^ The unfolded band structure of O_v_-Ti_3_C_2_O_2_ is shown in Figure S3. As shown in Figure S5, the photoabsorption spectrum of O_v_-Ti_3_C_2_O_2_ closely resembles that of pristine Ti_3_C_2_O_2_. Compared with pristine Ti_3_C_2_O_2_, O_v_-Ti_3_C_2_O_2_ retains a predominantly plasmonic mode at low energy, and the higher-energy feature still shows a mixed character. Except for the slightly shifted frequency (3.34 eV for Ti_3_C_2_O_2_ and 3.39 eV for O_v_-Ti_3_C_2_O_2_), all other laser pulse parameters for O_v_-Ti_3_C_2_O_2_ are identical to those used for pristine Ti_3_C_2_O_2_. The central frequency of 3.39 eV, instead of 1.51 eV, is chosen to drive CO_2_ dissociation on the surface as the unoccupied states of CO_2_ are located at higher energies as discussed below. As shown in Figure S6, the incident light pulse induces a pronounced dipole moment in O_v_-Ti_3_C_2_O_2_ at ∼10 fs. Subsequently, the excited electrons rapidly lose their collective coherence, resulting in a gradual attenuation of the dipole amplitude. As the plasmon decays, the occupation probabilities of hot carriers increase gradually (Figure S7) with excitations occurring predominantly from occupied states at ∼−0.83 eV to unoccupied states around 2.56 eV, similar to the pristine Ti_3_C_2_O_2_.

Before examining the catalytic performance of hot electrons, we first presented the thermodynamic and kinetic information on CO_2_ dissociation at the ground state. As shown in Figure 4a, CO_2_ binds weakly to the O_v_ site (adsorption energy is −0.65 eV) and maintains its linear configuration. The similar electronic DOS of O_v_-Ti_3_C_2_O_2_ before and after CO_2_ adsorption further indicates its weak adsorption for CO_2_ (Figure S8). The linear CO_2_ binds to the O_v_ site in a tilted end-on configuration, allowing σ-type interaction between the vacancy electrons and the CO_2_ *σ LUMO, which leads to a notable broadening of the LUMO compared to gas-phase CO_2_ (Figure 4b). The LUMO of free CO_2_, located at ∼4.3 eV, shifts below 4 eV upon adsorption (with broadened states extended even below 3 eV), suggesting that incident near-ultraviolet photons may be required to drive CO_2_ dissociation. As shown in Figure 4c, *CO_2_ dissociation into CO and lattice O on O_v_-Ti_3_C_2_O_2_ involves a kinetic barrier of 1.21 eV and a reaction energy of −0.7 eV, where partial occupation of the transition-state LUMO weakens the C–O bond, leading to its elongation and eventual cleavage (Figure S9).

**Figure 4:**
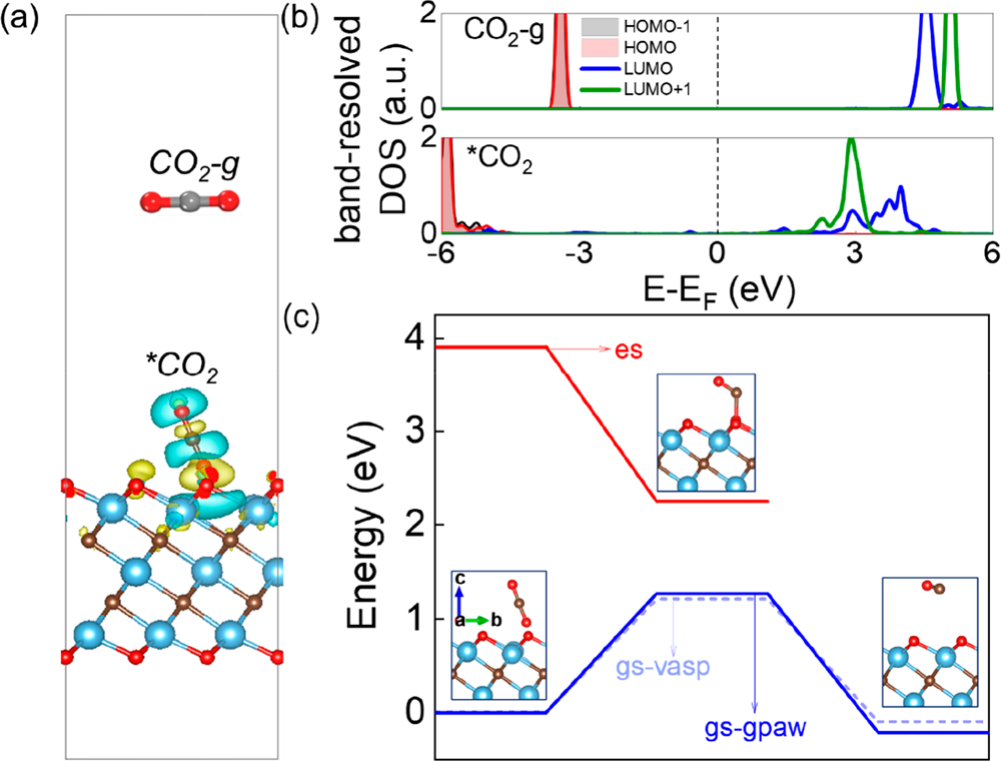
(a) Charge density difference of the CO_2_@O_v_-Ti_3_C_2_O_2_ adsorption system (CO_2_-g and *CO_2_ represent CO_2_ in the gas phase and adsorbed CO_2_, respectively). The yellow and blue regions indicate electron accumulation and depletion, respectively. (b) Band-resolved DOS of individual bands for CO_2_ gas and adsorbed CO_2_. (c) Energy profiles [ground (gs) and excited (es) states] and atomic structures of CO_2_ dissociation on O_v_-Ti_3_C_2_O_2_ and a comparison of GPAW (blue) and VASP (light blue) ground-state calculations are presented.

For CO_2_ dissociation assisted by hot electrons, we simulated the excitation of hot electrons around the Fermi level of O_v_-Ti_3_C_2_O_2_ into the LUMO of *CO_2_. As illustrated in Figure 4c, the ground-state energy of CO_2_ dissociation calculated using GPAW and VASP are consistent with each other. The excited CO_2_@O_v_-Ti_3_C_2_O_2_ state is constructed using the linear expansion ΔSCF method,^56^ which has been applied to study surface reactions involving excited or transiently charged states.^53,57–62^ Figure 4c shows that hot electron injected into the LUMO significantly reduces the kinetic barrier for CO_2_ dissociation from 1.21 eV to nearly 0 eV, indicating that dissociation into CO can readily occur with hot electron assistance. This reduction is driven by a largely reduced excitation energy at the transition state, due to elongation of the C–O bond in CO_2_ and downshifted LUMO (Figure S9). Given the limitations of GGA-DFT for interfacial level alignment and charge-transfer excitations, the computed excitation energy from ΔSCF is treated as semiquantitative. Notably, ΔSCF does not model hot-carrier statistics; it instead approximates the charge-transferred state by fully occupying the targeted unoccupied orbital, representing an idealized scenario that may not fully capture hot-carrier distributions under realistic conditions. The calculated excitation energy is 3.92 eV and provides an estimate of the relevant energetic requirement (see Figure S10 for more details of the large supercell).

The charge density difference (Figure S11) illustrates the electron distribution between the ground and excited states, which closely resembles that in Figure 4a; in both cases electrons are transferred around the Fermi level of O_v_-Ti_3_C_2_O_2_ to the broadened LUMO of *CO_2_. As discussed above, the hot electrons distribution is centered around ∼2.5 eV above the Fermi level under 3.39 eV irradiation. Therefore, we anticipate that the injection of hot electrons, partially occupying the broadened LUMO (tails extended to 2–3 eV above the Fermi energy), can still reduce the energy barrier for CO_2_ reduction. In addition, low-energy hot electrons can still promote reactions by transferring energy to adsorbate vibrational modes and/or through local photothermal heating.^63–66^ Furthermore, rt-TDDFT simulations^67–71^ show that CO_2_ dissociates; the resulting CO fragment becomes negatively charged transiently, ultimately forming on the Ti_3_C_2_O_2_ surface (see Figure S12 for details).

We further investigated different excitation channels and their impacts in the CO_2_ dissociation pathways. As shown in Figure S13, hot holes transfer from the Fermi level to the highest occupied molecular orbital (HOMO; or correspondingly electron excited from the HOMO to the Fermi level) of *CO_2_ requires a high excitation energy of 7.62 eV, reflecting the deep energy position of the HOMO at approximately −6 eV below the Fermi energy. Intramolecular HOMO–LUMO excitation within CO_2_ also requires substantial energy because of the large gap between HOMO and LUMO of *CO_2_ (Figure S14). Plasmon-mediated CO_2_ dissociation into CO is thus most likely driven by hot electrons, originating from excitation from the Fermi level of O_v_-Ti_3_C_2_O_2_ and injected into the LUMO of *CO_2_.

Next, we discuss the feasibility of forming these oxygen vacancies through H_2_ reduction, and our results indicate that O_v_ formation with H_2_ assistance is relatively challenging. On the pristine Ti_3_C_2_O_2_ surface, H_2_ binds weakly and undergoes homolytic scission, which requires a kinetic barrier of 1.96 eV (Figure S15). After H_2_ dissociation, the dissociated H atoms bond with surface O atoms to form OH groups. The formation of O_v_ then requires one H atom to cleave its O–H bond and react with another OH to form H_2_O, simultaneously producing the oxygen vacancy, with a kinetic barrier of 1.38 eV. As shown in Figure S16, we further investigated H_2_-induced O_v_ formation on the surface already containing an O_v_, evaluating whether the pre-existing oxygen defect facilitates the generation of additional vacancies. H_2_ preferentially adsorbs at the O_v_ site and dissociates, with one H occupying the O_v_ and the other binding to a nearby O atom to form an OH group, requiring a relatively low kinetic barrier of 0.65 eV. However, the subsequent O_v_ formation accompanied by H_2_O generation, via H transfer from the O_v_ site to the OH group, is hindered by a high energy barrier of 2.2 eV, due to the strong binding of H at the O_v_ site. In addition, we investigated H_2_ dissociation on the OH-functionalized surface to assess whether the presence of an OH group facilitates H_2_ dissociation and O_v_ formation (Figure S17). However, H_2_ dissociation and subsequent H_2_O formation on this surface remain unfavorable, exhibiting a high kinetic barrier of 2.65 eV. These results indicate that vacancy formation in Ti_3_C_2_O_2_ via H_2_ reduction is challenging. However, transition metals such as Pt or Pd can be introduced to the Ti_3_C_2_O_2_ surface to facilitate H_2_ dissociation. The dissociated H atoms may migrate to the surface and react with lattice oxygen to form H_2_O, leaving oxygen vacancies behind for CO_2_ dissociation, like in a reverse Mars-van Krevelen mechanism.^72–76^

Given the limitations of semilocal GGA-DFT in interfacial energy-level alignment, the single-particle component of the rt-TDDFT results can be sensitive to the underlying level alignment. For metallic Ti_3_C_2_O_2_, where the Ti *d* states are relatively delocalized, introducing Hubbard U^77^ significantly changes the calculated optical response and reduces the agreement with the measured absorption spectrum; therefore, *U* = 0 has been used in this work (Figure S18). More sophisticated many-body approaches can improve the interfacial level alignment and have been explored in hot-carrier injection modeling,^78–80^ but they remain computationally demanding for the present system and are left for future work.

In conclusion, we conducted a comprehensive theoretical study of the plasmonic properties of Ti_3_C_2_O_2_ within the framework of rt-TDDFT and examined the catalytic performance of hot carrier generated from plasmon decay for a probing reaction, CO_2_ dissociation at the O_v_-Ti_3_C_2_O_2_. Our results reveal that a laser pulse induces plasmon formation in Ti_3_C_2_O_2_ and O_v_-Ti_3_C_2_O_2_, characterized by evolution of the dipole moment. Notably, the excited plasmon decays rapidly, which can be attributed to the strong electron-magnetic absorption of Ti_3_C_2_O_2_. Low O_v_ concentrations have a minor effect on the plasmonic properties of Ti_3_C_2_O_2_ and mainly serve as reaction sites. The excited Ti_3_C_2_O_2_ and O_v_-Ti_3_C_2_O_2_ systems efficiently channel the stored energy from the laser pulse into hot-carrier generation. Linear expansion ΔSCF calculations reveal that hot electrons can enhance CO_2_ dissociation, eliminating the kinetic barrier on O_v_-Ti_3_C_2_O_2_. Additionally, we found that H_2_-assisted formation of O_v_ is unfavorable on the Ti_3_C_2_O_2_ surface but combination with transition metals is expected to facilitate the oxygen vacancy formation. Overall, these findings highlight the plasmonic characteristics of Ti_3_C_2_O_2_ and provide valuable insights into the reductive reaction pathways mediated by hot carriers on this emerging family of plasmonic materials.

## Supplementary Material



## Notes

Computational details and methods, convergence tests of the supercell size and plane-wave cutoff energy, band structures of Ti_3_C_2_O_2_ and O_v_-Ti_3_C_2_O_2_, TDDFT absorption spectra and dipole moment dynamics for Ag_20_ and Au_20_, absorption spectrum of O_v_-Ti_3_C_2_O_2_, electric field pulse and dipole moment oscillation for O_v_-Ti_3_C_2_O_2_, hot-carrier generation in O_v_-Ti_3_C_2_O_2_ at 10, 15, and 30 fs, pDOS of O_v_-Ti_3_C_2_O_2_ before and after CO_2_ adsorption, DOS of individual bands for the transition state of CO_2_ dissociation, construction of the relevant excited states, linear expansion ΔSCF calculation in the 5 × 5 × 1 supercell, charge density difference between the ground and excited states for the CO_2_@O_v_-Ti_3_C_2_O_2_ adsorption system, rt-TDDFT simulations of CO_2_ dissociation dynamics, linear expansion ΔSCF calculations for different hot-carrier-mediated reaction pathways, kinetic barriers and atomic structures of H_2_ dissociation via the homolytic pathway on Ti_3_C_2_O_2_ and the heterolytic pathway on O_v_-Ti_3_C_2_O_2_, kinetic barrier and atomic structures of H_2_O formation from H_2_ on OH-group-functionalized Ti_3_C_2_O_2_, TDDFT absorption spectra of Ti_3_C_2_O_2_ and O_v_-Ti_3_C_2_O_2_ computed using DFT+*U* (PDF)
