# Supplementary material for: Plasmonic Hot-Carrier Generation and Catalysis in Ti3C2O2 from Real-Time TDDFT Simulations
Source: Nano Lett. 2026 Apr 10;26(15):5229–35. doi: 10.1021/acs.nanolett.6c00650 (PMC13107522; doi:10.1021/acs.nanolett.6c00650)
Supplement: Supplementary file 1 [file nl6c00650_si_001.pdf]

Supporting Information for

Plasmonic Hot-Carrier Generation and Catalysis in $Ti_3C_2O_2$ from Real-Time TDDFT Simulations

*Na Zhang[1], Shoutian Sun[1], Bin Wang[1,2]**

1 School of Sustainable Chemical, Biological and Materials Engineering, University of Oklahoma, Norman, Oklahoma 73019, United States

2 Department of Chemical and Biological Engineering, Tufts University, Medford, Massachusetts 02155, United States

KEYWORDS: $Ti_3C_2O_2$, plasmonic catalysis, hot carrier, time-dependent density functional theory (TDDFT), localized surface plasmon resonance (LSPR), CO_2 reduction

Contents

Calculation details 3
Convergence tests of supercell size and Plane-Wave cutoff energy 8
The unfolded band structure of pristine $Ti_3C_2O_2$ and O_v-$Ti_3C_2O_2$ 9
Rt-TDDFT simulations of Ag_{20} and Au_{20} 10
The Plasmonic Study of O_v-$Ti_3C_2O_2$ 10
The photoabsorption spectrum 10
The dipole moment evolution of O_v-$Ti_3C_2O_2$ 11
Hot carrier generation of O_v-$Ti_3C_2O_2$ at different times 11
The density of states of O_v-$Ti_3C_2O_2$ before and after adsorption 12
Linear expansion ΔSCF calculation of CO_2 dissociation 13
Linear expansion ΔSCF calculation of a 5×5×1 supercell 14
Charge density difference between the ground and excited states 15
Rt-TDDFT simulations of CO_2 dissociation dynamics 15
Different hot carrier transfer channels in CO_2@O_v-$Ti_3C_2O_2$ adsorption system 16
The hot hole transfers to the HOMO of *CO_2 16
Intramolecular transfer between HOMO and LUMO of *CO_2 17
The O_v formation by the assistance of H_2 17
H_2 dissociation via homolytic scission on pristine $Ti_3C_2O_2$ 17
H_2 dissociation via heterolytic scission on O_v-$Ti_3C_2O_2$ 18
H_2 dissociation on OH-functionalized $Ti_3C_2O_2$ 19
TDDFT absorption spectrum using DFT+U 19
Reference 19

Calculation details

Real-time time dependent density functional theory (rt-TDDFT) was performed in the linear combination of atomic orbitals (LCAO) mode[1, 2] using the GPAW package[3, 4]. The current Kohn-Sham decomposition implementation in GPAW supports only Γ-point sampling (a 1×1×1 k-point mesh)[5]. Accordingly, the plasmonic properties of $Ti_3C_2O_2$ were evaluated using a 5×5×1 supercell with Γ-point-only sampling; the supercell-size convergence test is presented below. A vacuum slab of approximately 20 Å along the Z direction was included to prevent interlayer interaction. Ground-state calculations were carried out within DFT in GPAW (LCAO mode) using the Perdew-Burke-Ernzerhof (PBE) exchange-correlation functional[6-9] and a double-ζ polarized (dzp) basis set. A real space grid spacing of $h = 0.2$ Å was used for numerical integration. The self-consistent-field (SCF) convergence criterion for the electron density was set to 1×10^{-6} for structural relaxations and 1×10^{-12} for electronic structures calculations. Structural relaxations were performed with the BFGS optimizer as implemented in ASE until the maximum force on each atom was below 1×10^{-2} eV/Å[10]. Electronic occupations were treated using Fermi-Dirac smearing of 0.05 eV. For O_v-$Ti_3C_2O_2$, a 5×5 supercell with a single oxygen vacancy corresponds to ~4% defect concentration. This model is chosen to minimize defect-defect interaction while remaining computationally tractable.

TDDFT absorption spectrum. The TDDFT absorption spectrum was computed using the δ -kick method[11]. Specifically, an instantaneous electric field $\boldsymbol{E}(\boldsymbol{r},t) = E_0\hat{e}_z\delta(t)$ with $E_0 = 1 \times e^{-5}$ a.u. (~510 μV/Å) was applied along the Z-axis to perturb both

pristine $Ti_3C_2O_2$ and O_v-$Ti_3C_2O_2$. The field strength is sufficiently weak to ensure that the response between light and electrons remains in the linear regime. The absorption spectrum was obtained from the Fourier transform of the time-dependent dipole moment and broadened with a Gaussian function (σ = 0.07 eV). The plasmonic character of each excitation was quantified using the normalized plasmonicity index $\tilde{\eta}'_P$[12]. Specifically,

$$\tilde{\eta}'_P = \frac{\int |v_{ind}(\boldsymbol{r}, w_\xi)|^2 d^3\boldsymbol{r}}{\frac{f(w_\xi)}{w_\xi}},$$

where $v_{ind}(\boldsymbol{r}, w_\xi)$ is the induced potential generated by the induced chare density at the excitation frequency w_ξ. $f(w_\xi)$ is the oscillator strength of excitation ξ. A larger $\tilde{\eta}'_P$ value indicates a more collective (plasmonic) character of the corresponding absorption mode.

Plasmon dynamics simulations. To simulate the real time dynamics of localized surface plasmon resonance (LSPR), the system was excited by an incident light field polarized along the Z-axis, described by Gaussian pulse $\varepsilon(t) = \varepsilon_0 \cos(\omega_0(t - t_0))\exp(\frac{-(t-t_0)^2}{\tau_0^2})$. For $Ti_3C_2O_2$ and O_v-$Ti_3C_2O_2$, the central frequencies of the applied pulse were 3.34 eV and 3.39 eV, respectively. The pulse was centered t_0 = 10 fs, with a temporal width of τ_0 = 3 fs and a peak field amplitude of ~ 510 μV/Å. A time step of 0.01 fs and a total propagation time of 30 fs were employed.

Hot carrier generation analysis. For the hot carrier generation, we applied the convolution method developed by Tuomas P. Rossi, Paul Erhart, and Mikael Kuisma[13-16].. The transition contribution map (TCM) was used to visualize the contributions of

Kohn-Sham electron-hole transitions to photoabsorption[5]. In the TCMs, the diagonal line shows Kohn-Sham transitions whose eigenvalue difference match the excitation energy ω. The color intensity reflects the contribution magnitude, where darker colors correspond to stronger contributions.

Transition state calculations. Transition state search for CO_2 dissociation and H_2 dissociation were performed using the climbing-image nudged elastic band (CI-NEB) method[17]. A 3×3×1 supercell with ~20 Å of vacuum along the Z direction was used, and the Brillouin zone was sampled using a Γ-centered 3×3×1 k-point grid. The CI-NEB calculations were carried out using the projector augmented-wave (PAW) method[18] as implemented in the Vienna *Ab initio* Simulation Package (VASP)[19, 20], with the Perdew-Burke-Ernzerhof (PBE) exchange-correlation functional[6, 21]. A plane-wave cutoff energy of 450 eV was used, and the cutoff energy convergence test is presented below. For geometry optimization, the adsorbate and O_v-$Ti_3C_2O_2$ were allowed to move freely in all directions until the force on each atom was reduced to less than 1×10^{-2} eV/Å, and the energy variation between successive self-consistent steps was less than 10^{-5} eV.

The linear expansion ΔSCF calculations. Excited states were modeled by linear expansion ΔSCF calculations in GPAW (PW mode) with explicit occupation constraints on selected Kohn-Sham states[22]. Calculations were performed using a 3×3×1 supercell with a 3×3×1 k-point mesh, consistent with the CI-NEB calculations for CO_2 dissociation. In present study, both hot-electron and hot-hole transfer processes were evaluated via ΔSCF calculation. For hot-electron transfer, the excitation corresponds to

the transfer of one electron from an occupied state near the Fermi level of $Ti_3C_2O_2$ to the lowest unoccupied molecular orbital (LUMO) of the adsorbed CO_2. For hot-hole transfer, the excitation corresponds to the transfer of one hole from $Ti_3C_2O_2$ to the highest occupied molecular orbital (HOMO) of the adsorbed CO_2, which can be equivalently described as the transfer of a hot electron from HOMO of CO_2 to $Ti_3C_2O_2$, that is, one electron is removed from HOMO of CO_2 and transferred to $Ti_3C_2O_2$ to simulate hot-hole transfer process. In these procedures, the adsorbed CO_2 molecule was treated as a separate fragment within the full CO_2@O_v-$Ti_3C_2O_2$ adsorption system, and its LUMO and HOMO were identified based on the orbital projection onto this fragment. For intra-molecular electron transfer within CO_2 (from HOMO to LUMO), the excitation energy can be equivalently described as the total energy of an electron transferred from HOMO of CO_2 to $Ti_3C_2O_2$ substrate, followed by an electron transferred from $Ti_3C_2O_2$ to LUMO of CO_2. The excited state constructed from the converged ground-state wave functions. The excitation energy was obtained as the total energy difference between the constrained excited state and the ground state, $\Delta E = E_{excited} - E_{ground}$.

Simulation for CO_2 dissociation using rt-TDDFT. The laser pulse for real-time TDDFT simulation is described by a monochromatic Gaussian light pulse applied perpendicular to the CO_2@O_v-$Ti_3C_2O_2$ surface along the *z*-direction. The electric field of light pulse is described as $\varepsilon(t) = \varepsilon_0 \cos(\omega_0(t - t_0)) \exp(-(t - t_0)^2/\tau_0^2)$, where ε_0 is the maximum field strength of 2.5 V/Å, ω_0 is the central frequency of 3.39 eV, t_0 and τ_0 is the centered time of 30 fs and Gaussian pulse width 2.97 fs (FWHM =

$2\sqrt{2\ln\ 2}\tau_0$, FWHM = 7 fs). Time-dependent charge analysis was performed using the Hirshfeld charge scheme. The PBE exchange-correlation functional[21] was used with a mesh cutoff of 120 Ry, as implemented in TDAP code[23-26]. A double-ζ polarized (dzp) basis set was used, and Fermi smearing was set with an electronic temperature of 300 K. Norm-conserving pseudopotentials were obtained from ABINIT's Fritz-Haber-Institute pseudo database for all atoms. Nonadiabatic molecular dynamics is treated within the Ehrenfest scheme, where the ground state is used as initial state. The total propagation time is 65 fs, with a time step of 0.02 fs.

Convergence tests of supercell size and Plane-Wave cutoff energy

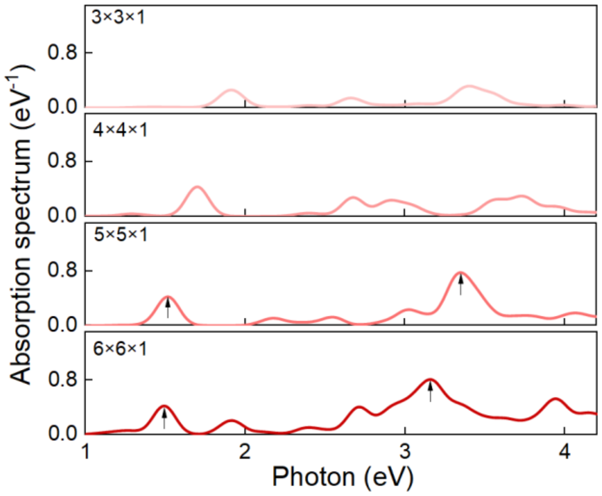

Figure S1. Convergence test of the supercell size for TDDFT absorption spectra of $Ti_3C_2O_2$.

Spectra computed using 3×3×1, 4×4×1, and 5×5×1 supercells exhibit finite-size effects, which decrease systematically with increasing cell size and are largely suppressed for 6×6×1. The spectra obtained with 5×5×1 and 6×6×1 supercells are in close agreement. In particular, the 1.5 eV peak matches very well, and the higher-energy peak differs by ~5-6% (3.34 eV for 5×5×1 vs 3.16 eV for 6×6×1). Accordingly, we adopted a 5×5×1 supercell as a practical compromise between accuracy and computational cost.

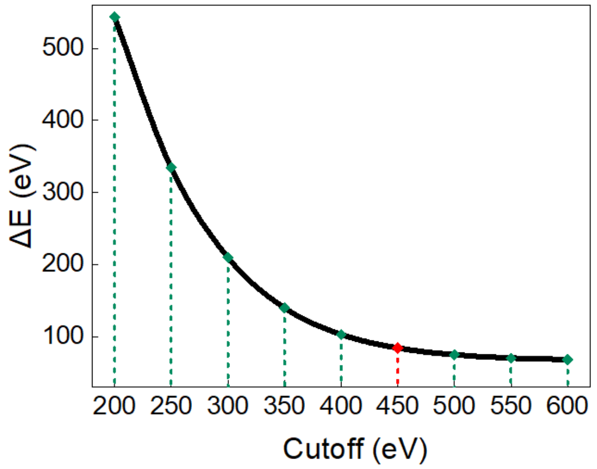

Figure S2. Convergence test of the plane-wave cutoff energy for linear expansion ΔSCF calculations.

A cutoff energy of 450 eV was selected at which the total energy of the system is well converged. The cutoff test was performed using self-consistent field calculations in GPAW for the optimized 3×3×1 supercell, consistent with the subsequent PW-mode ΔSCF calculations.

The unfolded band structure of pristine $Ti_3C_2O_2$ and O_v-$Ti_3C_2O_2$

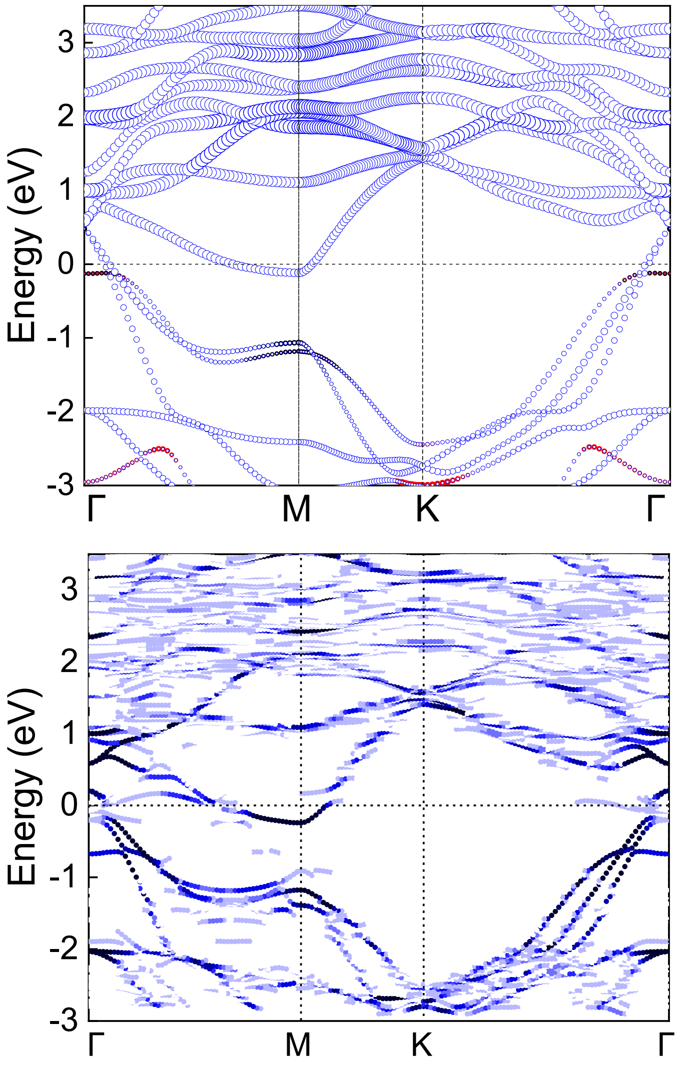

Figure S3. The unfold band structure of $Ti_3C_2O_2$ (upper panel) and O_v-$Ti_3C_2O_2$ (lower panel).

Rt-TDDFT simulations of Ag_{20} and Au_{20}

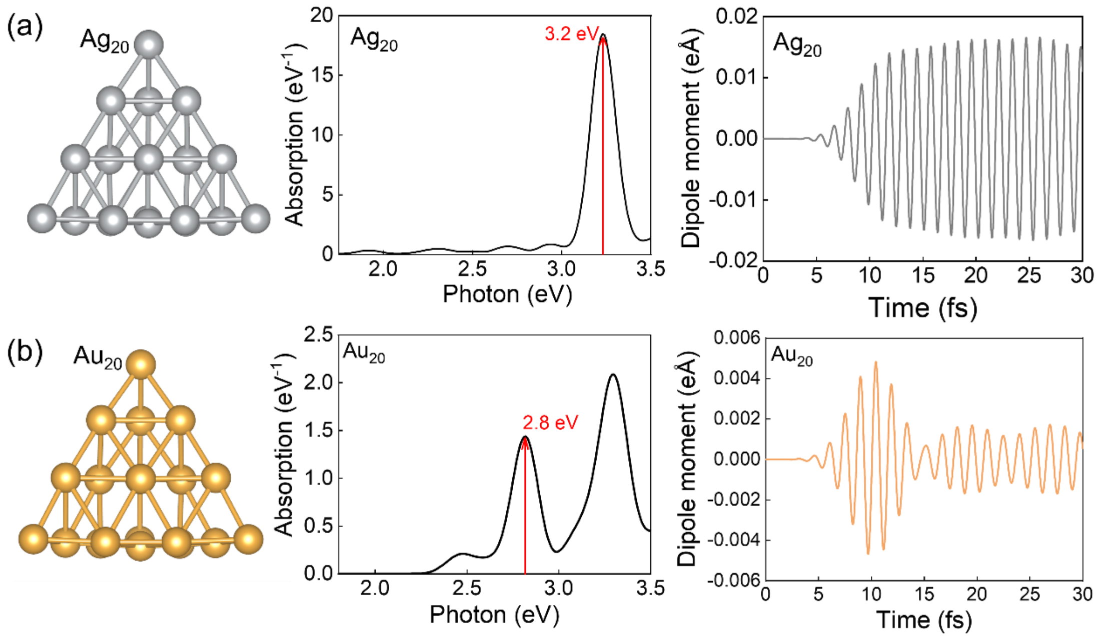

Figure S4. Atomic structure, TDDFT absorption spectrum, and time-dependent dipole moments of (a) Ag_{20} and (b) Au_{20}. The δ-kick (infinite-frequency) pulse and the single-frequency pulse are applied along the *z*-axis. Except for the laser frequency determined from the absorption spectrum, all other pulse parameters are the same as those used for $Ti_3C_2O_2$.

The plasmon energies of Ag_{20} and Au_{20} are calculated to be ~3.2 and ~2.8 eV, respectively, in good agreement with the reported values (~3.2 eV for Ag_{20} and ~2.78 eV for Au_{20})[12, 27].

The Plasmonic Study of O_v-$Ti_3C_2O_2$

The photoabsorption spectrum

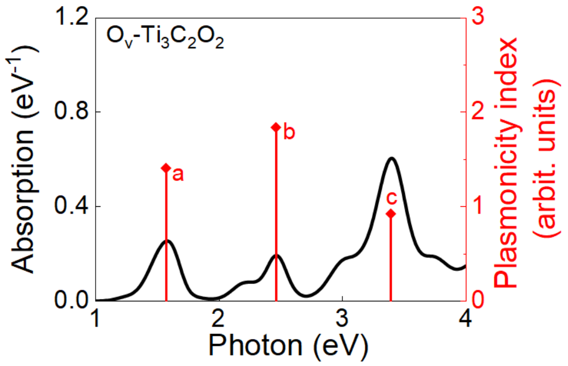

Figure S5. Optical absorption spectrum of O_V-$Ti_3C_2O_2$ (black) and the corresponding plasmonicity index (red diamonds; vertical lines mark peak positions).

The dipole moment evolution of O_v-$Ti_3C_2O_2$

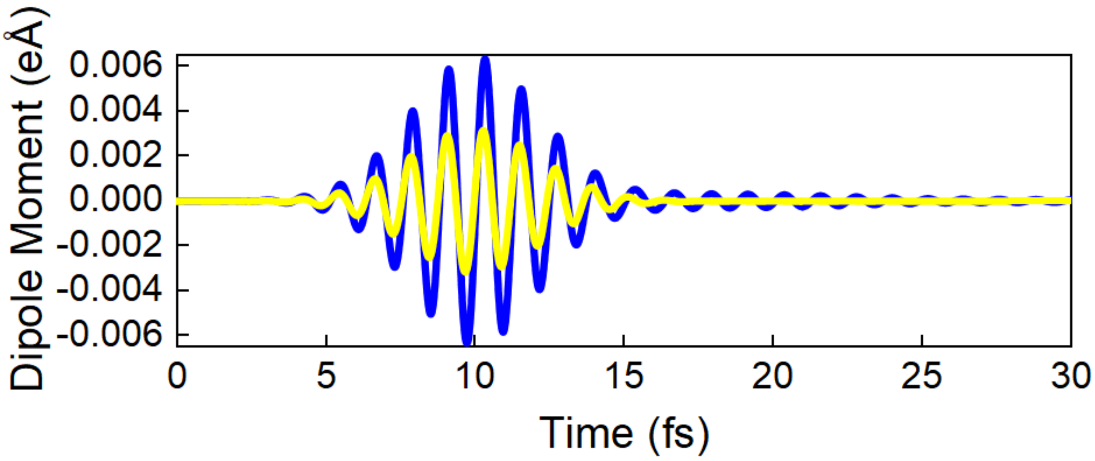

Figure S6. Electric field pulse (yellow curve) applied to excite O_v-$Ti_3C_2O_2$. The plasmon response (blue curve), is characterized by the time-dependent dipole moment of O_v-$Ti_3C_2O_2$.

Hot carrier generation of O_v-$Ti_3C_2O_2$ at different times

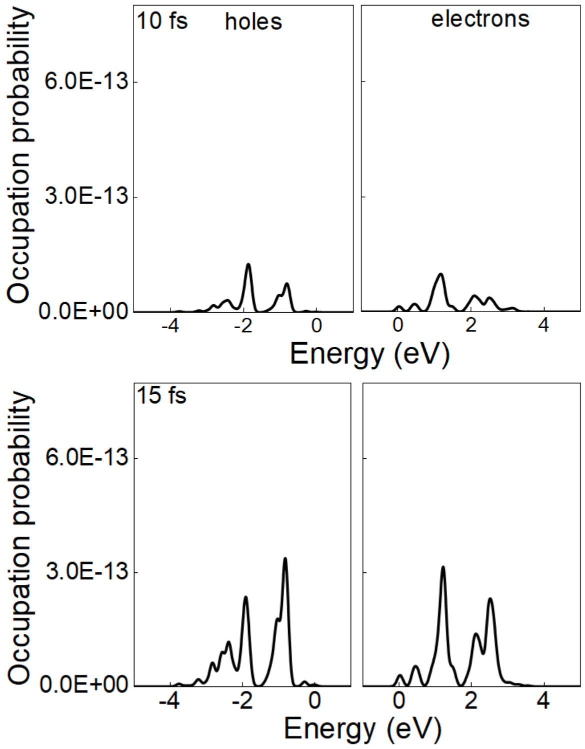

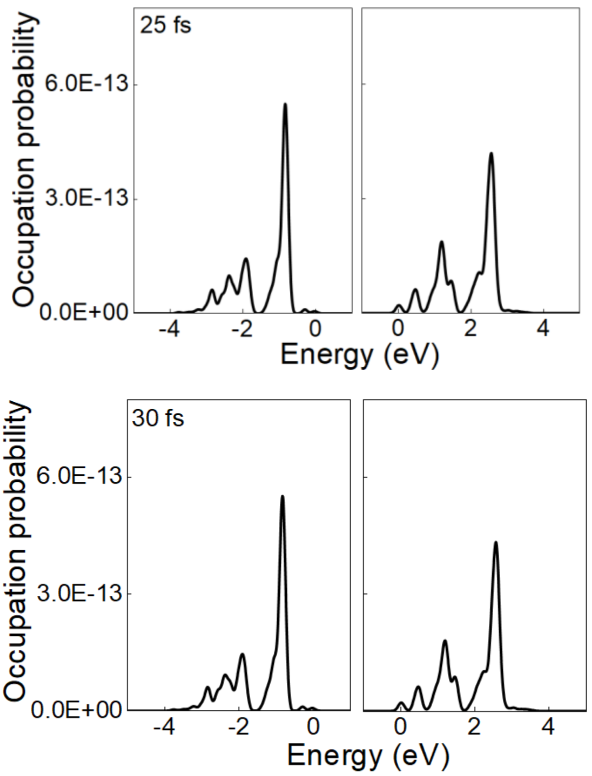

Figure S7. Time evolution of hot-electron and hot-hole occupations in O_v-$Ti_3C_2O_2$ at 10, 15, 25, and 30 fs.

The density of states of O_v-$Ti_3C_2O_2$ before and after adsorption

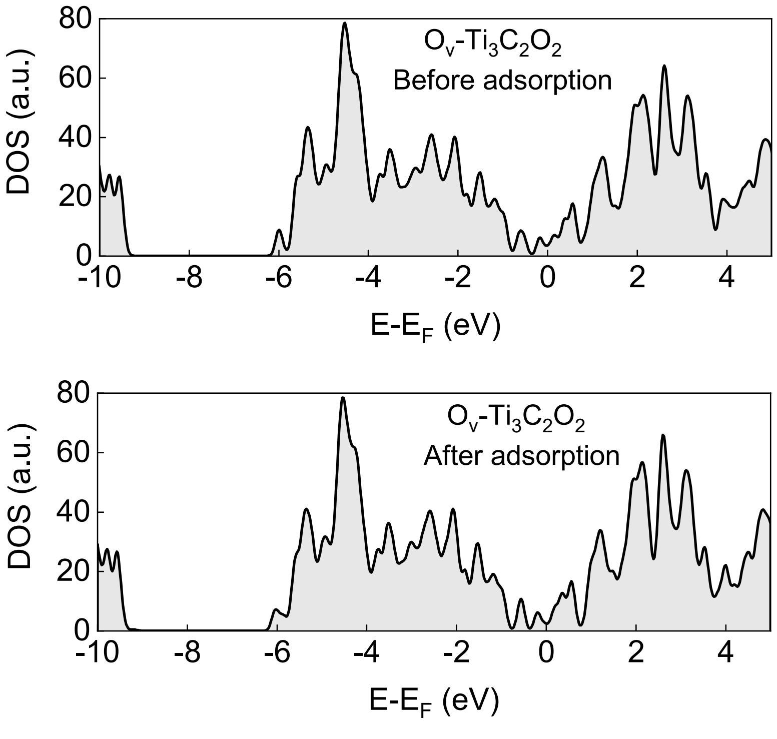

Figure S8. DOS of O_v-$Ti_3C_2O_2$ before and after CO_2 adsorption.

Linear expansion ΔSCF calculation of CO_2 dissociation

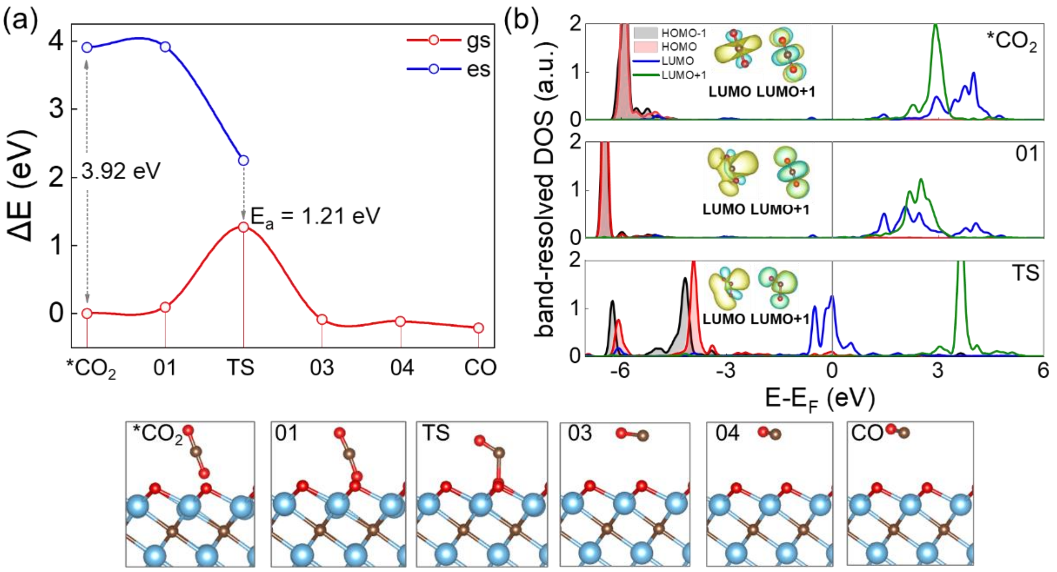

Figure S9. (a) Energy profiles of the ground and excited states. The numbers (01, 03, and 04) in the figure correspond to images along the NEB path. (b) The band-resolved DOS and orbital iso-surfaces of CO_2 moiety for $*CO_2$, NEB image (01), and TS structures along the NEB path.

In linear expansion ΔSCF calculations, the excited-state occupation is imposed on a CO_2-localized lowest unoccupied state. For $*CO_2$ and the early NEB image (01), the CO_2 unit remains (nearly) linear, and its frontier unoccupied orbital is a σ* state, so the target state for electron transfer can be straightforwardly identified from the CO_2 fragment. For the transition state, although CO_2 is distorted and the frontier orbitals may hybridize with O_v-$Ti_3C_2O_2$, we selected the lowest-energy unoccupied state with dominant CO_2 character (validated by CO_2-projected DOS and real-space orbital iso-surfaces) as the ΔSCF target state for electron transfer.

Linear expansion ΔSCF calculation of a 5×5×1 supercell

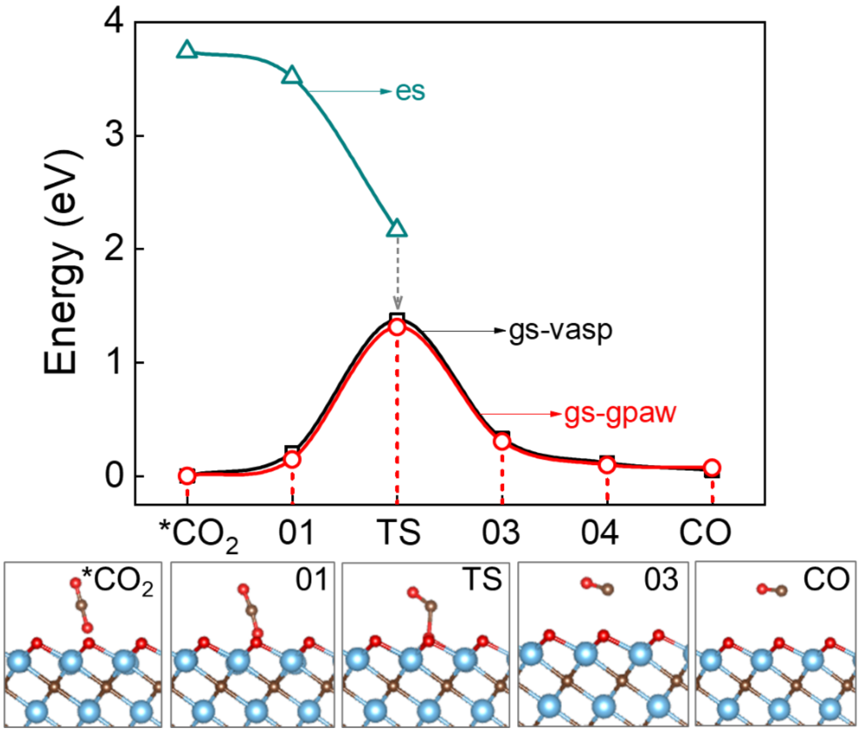

Figure S10. Energy profiles of the ground and excited states, and atomic structures along the NEB path for CO_2 dissociation on O_v-$Ti_3C_2O_2$ with a 5×5×1 supercell. Ground-state results obtained with GPAW (black curve) are compared with those from VASP (red curve).

CI-NEB calculation of CO_2 dissociation was performed on a 5×5×1 supercell using Γ-point sampling (1×1×1). The corresponding linear expansion ΔSCF excited states were then constructed from the converged 5×5×1 ground state configurations to form a state-specific interfacial charge-transfer sate (O_v-$Ti_3C_2O_2$ → CO_2). In the 5×5×1 supercell, the resulting ΔSCF charge-transfer excitation energy is 3.73 eV, which is closer to the plasmon-resonant excitation window than 3×3×1. These results lead to the same conclusion as in the 3×3×1 supercell: plasmon-generated hot electrons can facilitate CO_2 dissociation.

Charge density difference between the ground and excited states

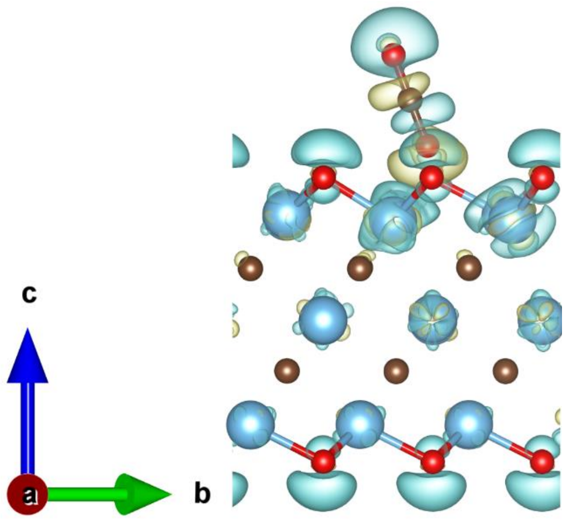

Figure S11. Charge density difference between the ground and excited states for CO_2@O_v-$Ti_3C_2O_2$ adsorption system. The yellow and blue regions indicate electron accumulation and depletion, respectively.

Rt-TDDFT simulations of CO_2 dissociation dynamics

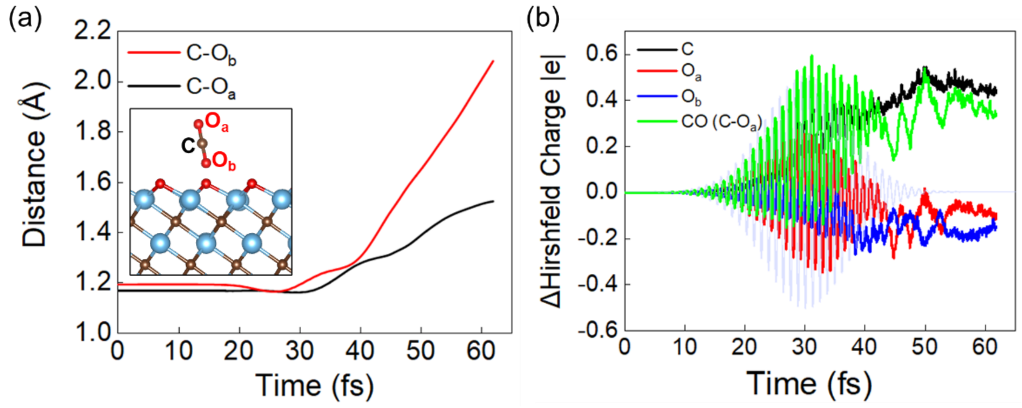

Figure S12. (a) Time evolution of the C-O_a and C-O_b bond distances. (b) Time dependent Δ Hirshfeld Charge of C, O_a, O_b, and CO_a moiety in CO_2. Positive and negative Δ Hirshfeld Charge values indicate electron accumulation and depletion relative to the initial state, respectively. The light blue Gaussian-shaped curve represents the applied laser field, which is centered at 30 fs. In (a) and (b), the simulation time is 65 fs with a time step of 0.02 fs.

The CO_2 dissociation dynamics were simulated using real-time TDDFT. Upon application of an external laser pulse with a frequency of 3.39 eV and a peak electric field strength of 2.5 V/Å centered at 30 fs, the CO_2 molecule responds rapidly. As shown in Figure S12a, the C-O_b bond elongates more significantly than the C-O_a bond and

eventually breaks. Concurrently, the O_b atom migrates to the vacancy site, while the remaining fragment forms a CO molecule on the surface. Time-dependent Δ Hirshfeld charges of the CO_2 moiety illustrate the evolution of charge distribution. As shown in Figure S12b, during the dissociation process, the charge on O_a and O_b exhibits oscillatory behavior and gradually decreases, while the C atom gains electrons from $Ti_3C_2O_2$. Consequently, a transient electron-rich CO (C-O_a) is formed on the surface. Such rapid CO response and the accompanying electron redistribution suggests that direct interfacial charge transfer may be involved in this reaction under the present conditions.

Different hot carrier transfer channels in CO_2@O_v-$Ti_3C_2O_2$ adsorption system

The hot hole transfers to the HOMO of *CO_2

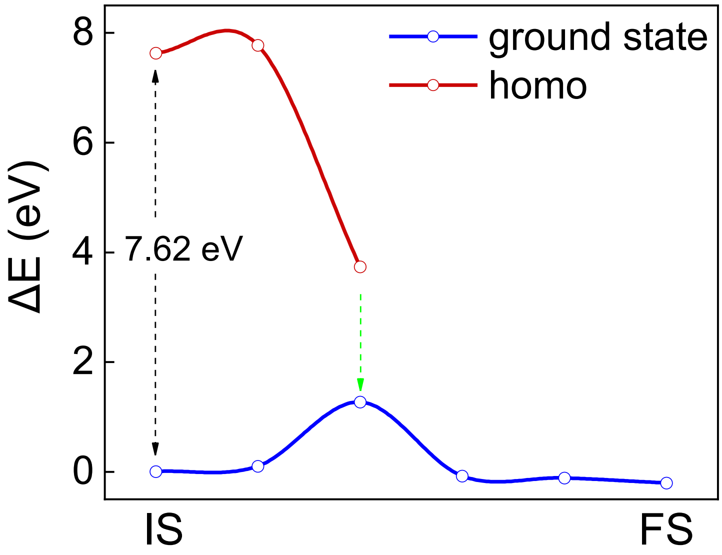

Figure S13. Energy profile of the ground and excited states of CO_2 dissociation into CO. The hot hole transfers from the Fermi level of O_v-$Ti_3C_2O_2$ to the HOMO of *CO_2.

Intramolecular transfer between HOMO and LUMO of $*CO_2$

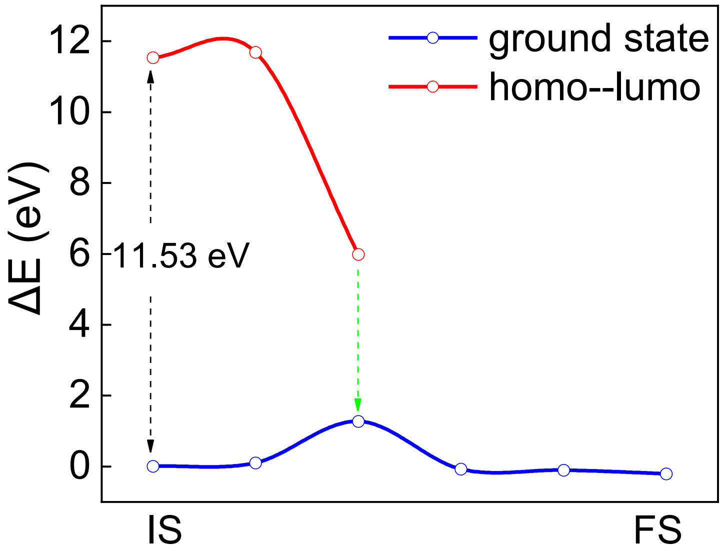

Figure S14. Energy profile of the ground and excited states of CO_2 dissociation into CO. The electron transfers from HOMO to LUMO of $*CO_2$.

The O_v formation by the assistance of H_2

H_2 dissociation via homolytic scission on pristine $Ti_3C_2O_2$

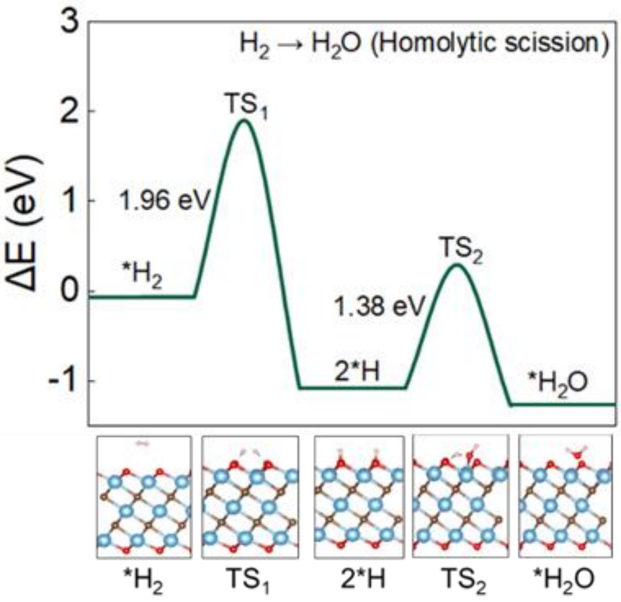

Figure S15. The O_v formation via H_2 assistance. The energy profile of H_2 reduction as H_2O on the pristine $Ti_3C_2O_2$. The atomic structures correspond to the initial, transition, and final states along the NEB reaction pathway.

H_2 dissociation via homolytic scission on pristine $Ti_3C_2O_2$, consisting of two subprocesses. The first involves H_2 dissociation at the pristine surface, for which five intermediate images were inserted. The second corresponds to H transfer to the neighboring OH, resulting in H_2O formation and the creation of an oxygen vacancy; five intermediate images were also inserted.

H_2 dissociation via heterolytic scission on O_v-$Ti_3C_2O_2$

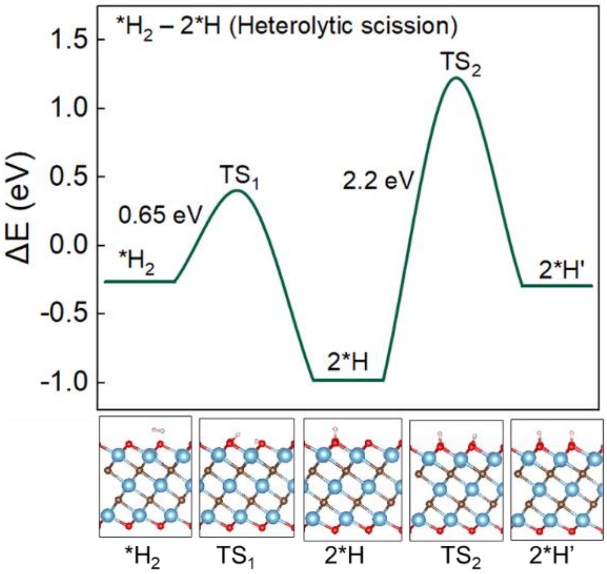

Figure S16. The O_v formation via H_2 assistance. The energy profile of H_2 reduction as H_2O on the defect $Ti_3C_2O_2$ (the surface with an O_v). The atomic structures correspond to the initial, transition, and final states along the NEB path.

H_2 dissociation via heterolytic scission on defective $Ti_3C_2O_2$ with one oxygen vacancy, consisting of two subprocesses. The first involves H_2 dissociation at the vacancy site, for which four intermediate images were inserted. The second corresponds to H transfer from the oxygen vacancy to a surface O atom, with four intermediate images were also used.

H_2 dissociation on OH-functionalized $Ti_3C_2O_2$

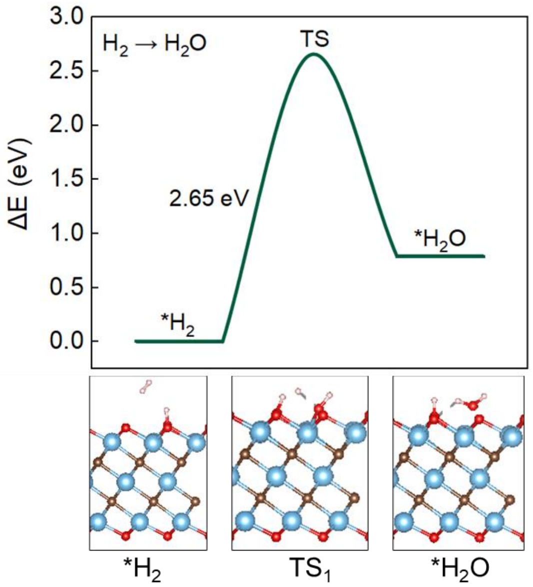

Figure S17. H_2 dissociation on OH-functionalized $Ti_3C_2O_2$. Four intermediate images were inserted for searching the transition state. The atomic structures correspond to the initial, transition, and final states along the NEB path.

TDDFT absorption spectrum using DFT+U

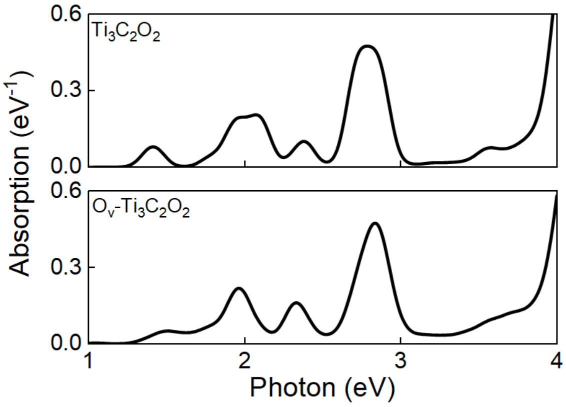

Figure S18. TDDFT absorption spectra of $Ti_3C_2O_2$ and O_v-$Ti_3C_2O_2$ computed using DFT+U. The Hubbard U value of 5.12 eV was used[28].

Reference

1. Kuisma, M., et al., *Localized surface plasmon resonance in silver nanoparticles: Atomistic first-principles time-dependent density-functional theory calculations.* Physical Review B, 2015. **91**(11): p. 115431.
2. Runge, E. and E.K.U. Gross, *Density-Functional Theory for Time-Dependent Systems.*

Physical Review Letters, 1984. **52**(12): p. 997-1000.

3. Enkovaara, J., et al., *Electronic structure calculations with GPAW: a real-space implementation of the projector augmented-wave method.* Journal of Physics: Condensed Matter, 2010. **22**(25): p. 253202.
4. Mortensen, J.J., L.B. Hansen, and K.W. Jacobsen, *Real-space grid implementation of the projector augmented wave method.* Physical Review B, 2005. **71**(3): p. 035109.
5. Rossi, T.P., et al., *Kohn–Sham Decomposition in Real-Time Time-Dependent Density-Functional Theory: An Efficient Tool for Analyzing Plasmonic Excitations.* Journal of Chemical Theory and Computation, 2017. **13**(10): p. 4779-4790.
6. Perdew, J.P., M. Ernzerhof, and K. Burke, *Rationale for mixing exact exchange with density functional approximations.* The Journal of Chemical Physics, 1996. **105**(22): p. 9982-9985.
7. Hohenberg, P. and W. Kohn, *Inhomogeneous Electron Gas.* Physical Review, 1964. **136**(3B): p. B864-B871.
8. Larsen, A.H., et al., *Localized atomic basis set in the projector augmented wave method.* Physical Review B, 2009. **80**(19): p. 195112.
9. Kohn, W. and L.J. Sham, *Self-Consistent Equations Including Exchange and Correlation Effects.* Physical Review, 1965. **140**(4A): p. A1133-A1138.
10. Hjorth Larsen, A., et al., *The atomic simulation environment—a Python library for working with atoms.* Journal of Physics: Condensed Matter, 2017. **29**(27): p. 273002.
11. Yabana, K. and G.F. Bertsch, *Time-dependent local-density approximation in real time.* Physical Review B, 1996. **54**(7): p. 4484-4487.
12. Bursi, L., et al., *Quantifying the Plasmonic Character of Optical Excitations in Nanostructures.* ACS Photonics, 2016. **3**(4): p. 520-525.
13. Rossi, T.P., P. Erhart, and M. Kuisma, *Hot-Carrier Generation in Plasmonic Nanoparticles: The Importance of Atomic Structure.* ACS Nano, 2020. **14**(8): p. 9963-9971.
14. Fojt, J., et al., *Tailoring Hot-Carrier Distributions of Plasmonic Nanostructures through Surface Alloying.* ACS Nano, 2024. **18**(8): p. 6398-6405.
15. Fojt, J., et al., *Hot-Carrier Transfer across a Nanoparticle–Molecule Junction: The Importance of Orbital Hybridization and Level Alignment.* Nano Letters, 2022. **22**(21): p. 8786-8792.
16. Kumar, P.V., et al., *Direct hot-carrier transfer in plasmonic catalysis.* Faraday Discussions, 2019. **214**(0): p. 189-197.
17. Henkelman, G., B.P. Uberuaga, and H. Jónsson, *A climbing image nudged elastic band method for finding saddle points and minimum energy paths.* The Journal of Chemical Physics, 2000. **113**(22): p. 9901-9904.
18. Kresse, G. and D. Joubert, *From ultrasoft pseudopotentials to the projector augmented-wave method.* Physical Review B, 1999. **59**(3): p. 1758-1775.
19. Kresse, G. and J. Furthmüller, *Efficiency of ab-initio total energy calculations for metals and semiconductors using a plane-wave basis set.* Computational Materials Science, 1996. **6**(1): p. 15-50.
20. Kresse, G. and J. Furthmüller, *Efficient iterative schemes for ab initio total-energy calculations using a plane-wave basis set.* Physical Review B, 1996. **54**(16): p. 11169-

11186.

21. Perdew, J.P., K. Burke, and M. Ernzerhof, *Generalized Gradient Approximation Made Simple.* Physical Review Letters, 1996. **77**(18): p. 3865-3868.
22. Gavnholt, J., et al., *$\ensuremath{\Delta}$ self-consistent field method to obtain potential energy surfaces of excited molecules on surfaces.* Physical Review B, 2008. **78**(7): p. 075441.
23. Jun, R., K. Efthimios, and M. Sheng, *Optical properties of clusters and molecules from real-time time-dependent density functional theory using a self-consistent field*. 2010, Taylor & Francis. p. 1829-1844.
24. Lian, C., et al., *Photoexcitation in Solids: First-Principles Quantum Simulations by Real-Time TDDFT (Adv. Theory Simul. 8/2018).* Advanced Theory and Simulations, 2018. **1**(8): p. 1870018.
25. Ma, W., et al., *Recent progresses in real-time local-basis implementation of time dependent density functional theory for electron–nucleus dynamics.* Computational Materials Science, 2016. **112**: p. 478-486.
26. Kolesov, G., et al., *Real-Time TD-DFT with Classical Ion Dynamics: Methodology and Applications.* Journal of Chemical Theory and Computation, 2016. **12**(2): p. 466-476.
27. Wu, X., et al., *Molecular dynamics study of plasmon-mediated chemical transformations††Electronic supplementary information (ESI) available.* Chemical Science, 2023. **14**(18): p. 4714-4723.
28. Bennett, J.W., et al., *A systematic determination of hubbard U using the GBRV ultrasoft pseudopotential set.* Computational Materials Science, 2019. **170**: p. 109137.
